# Supplementary material for: Deletion of wfs1 Impairs Oligodendrocyte Precursor Cells Dorsal Distribution and Myelination Through the wfs1-hmgcs1 Axis in Zebrafish
Source: Biology (Basel). 2026 Aug 16;15(16):1402. doi: 10.3390/biology15161402 (PMC13510151; doi:10.3390/biology15161402)
Supplement: Supplementary file 1 [file biology-15-01402-s001.zip › File S1-Transcriptome sequencing data and differentially expressed genes (DEGs) in wfs1-- zebrafish at 5 dpf/2.KEGG/wfs1KOvsWT/GSEA/WT/BASE_EXCISION_REPAIR/BASE_EXCISION_REPAIR(DRE03410).html]

Details for gene set BASE\_EXCISION\_REPAIR(DRE03410)[GSEA]

|  || Dataset | deg.sample |
| Phenotype | sample.cls |
| Upregulated in class | WT |
| GeneSet | BASE\_EXCISION\_REPAIR(DRE03410) |
| Enrichment Score (ES) | -0.31417662 |
| Normalized Enrichment Score (NES) | -1.2823172 |
| Nominal p-value | 0.1437247 |
| FDR q-value | 0.5150367 |
| FWER p-Value | 0.987 |
Table: GSEA Results Summary

  

Fig 1: Enrichment plot: BASE\_EXCISION\_REPAIR(DRE03410)      
 Profile of the Running ES Score & Positions of GeneSet Members on the Rank Ordered List

  

| PROBE | DESCRIPTION (from dataset) | GENE SYMBOL | GENE\_TITLE | RANK IN GENE LIST | RANK METRIC SCORE | RUNNING ES | CORE ENRICHMENT || 1 | 110438871 | LOC110438871 |  |  | 575 | 2.799 | 0.0636 | No |
| 2 | 393774 | pole3 |  |  | 941 | 2.179 | 0.1157 | No |
| 3 | 556995\_1 | lig1 |  |  | 956 | 2.157 | 0.1781 | No |
| 4 | 406730 | apex1 |  |  | 3117 | 1.008 | 0.1397 | No |
| 5 | 100008368 | nthl1 |  |  | 3677 | 0.897 | 0.1483 | No |
| 6 | 570901 | smug1 |  |  | 4432 | 0.756 | 0.1467 | No |
| 7 | 556456\_1 | pold1 |  |  | 4702 | 0.718 | 0.1591 | No |
| 8 | 492463 | neil3 |  |  | 5165 | 0.659 | 0.1639 | No |
| 9 | 100037336 | rfc1 |  |  | 5563 | 0.631 | 0.1698 | No |
| 10 | 393949 | unga |  |  | 7114 | 0.481 | 0.1352 | No |
| 11 | 558045 | parp4 |  |  | 8200 | 0.415 | 0.1132 | No |
| 12 | 554204 | polb |  |  | 8499 | 0.403 | 0.1156 | No |
| 13 | 445480 | xrcc1 |  |  | 8509 | 0.402 | 0.1270 | No |
| 14 | 259256\_1 | rfc3 |  |  | 8868 | 0.377 | 0.1268 | No |
| 15 | 259256 | rfc3 |  |  | 9590 | 0.327 | 0.1136 | No |
| 16 | 492463\_1 | neil3 |  |  | 10149 | 0.293 | 0.1047 | No |
| 17 | 563276 | lig3 |  |  | 10553 | 0.269 | 0.0999 | No |
| 18 | 558045\_1 | parp4 |  |  | 10679 | 0.262 | 0.1036 | No |
| 19 | 192320 | pole2 |  |  | 10741 | 0.259 | 0.1092 | No |
| 20 | 406435 | rfc4 |  |  | 10835 | 0.253 | 0.1137 | No |
| 21 | 692256 | pold3 |  |  | 11051 | 0.241 | 0.1139 | No |
| 22 | 558951 | tdg.2 |  |  | 11149 | 0.236 | 0.1178 | No |
| 23 | 566516 | mpg |  |  | 11761 | 0.203 | 0.1045 | No |
| 24 | 100150924 | polg |  |  | 12181 | 0.180 | 0.0966 | No |
| 25 | 559134 | parga |  |  | 12511 | 0.164 | 0.0910 | No |
| 26 | 560788 | parp1 |  |  | 13023 | 0.139 | 0.0790 | No |
| 27 | 393253 | neil1 |  |  | 13521 | 0.114 | 0.0668 | No |
| 28 | 556456 | pold1 |  |  | 13726 | 0.106 | 0.0634 | No |
| 29 | 796456 | pold2 |  |  | 13771 | 0.103 | 0.0651 | No |
| 30 | 556995 | lig1 |  |  | 14356 | 0.076 | 0.0489 | No |
| 31 | 386707 | fen1 |  |  | 14956 | 0.049 | 0.0315 | No |
| 32 | 553789 | tdg.1 |  |  | 15014 | 0.046 | 0.0311 | No |
| 33 | 101887128 | mbd4 |  |  | 15029 | 0.045 | 0.0319 | No |
| 34 | 100007906\_1 | parp2 |  |  | 15576 | 0.018 | 0.0153 | No |
| 35 | 571834 | si:dkey-28b4.7 |  |  | 16805 | -0.027 | -0.0225 | No |
| 36 | 445385 | rfc5 |  |  | 16927 | -0.032 | -0.0254 | No |
| 37 | 553610 | pole4 |  |  | 17453 | -0.052 | -0.0404 | No |
| 38 | 550274 | zgc:110269 |  |  | 17476 | -0.053 | -0.0395 | No |
| 39 | 335495 | parp3 |  |  | 17558 | -0.056 | -0.0404 | No |
| 40 | 569462\_1 | pnkp |  |  | 17626 | -0.059 | -0.0408 | No |
| 41 | 553405 | pole |  |  | 18294 | -0.089 | -0.0591 | No |
| 42 | 558403 | mutyh |  |  | 18475 | -0.097 | -0.0620 | No |
| 43 | 30678 | pcna |  |  | 18983 | -0.121 | -0.0744 | No |
| 44 | 795095 | hmgb1b |  |  | 19300 | -0.135 | -0.0804 | No |
| 45 | 571301 | ungb |  |  | 20227 | -0.176 | -0.1043 | No |
| 46 | 550335 | zgc:110224 |  |  | 20695 | -0.198 | -0.1132 | No |
| 47 | 503748 | rfc2 |  |  | 22293 | -0.284 | -0.1551 | No |
| 48 | 100150674 | polg2 |  |  | 22333 | -0.286 | -0.1480 | No |
| 49 | 405797 | aptx |  |  | 22678 | -0.306 | -0.1498 | No |
| 50 | 406697 | poll |  |  | 24588 | -0.420 | -0.1975 | No |
| 51 | 558198 | si:ch211-141o9.10 |  |  | 25987 | -0.528 | -0.2261 | No |
| 52 | 791194 | ogg1 |  |  | 28793 | -0.877 | -0.2886 | Yes |
| 53 | 796446\_1 | adprhl2 |  |  | 29248 | -0.989 | -0.2740 | Yes |
| 54 | 553405\_1 | pole |  |  | 30393 | -1.444 | -0.2678 | Yes |
| 55 | 558198\_1 | si:ch211-141o9.10 |  |  | 30718 | -1.689 | -0.2287 | Yes |
| 56 | 556995\_2 | lig1 |  |  | 30840 | -1.799 | -0.1801 | Yes |
| 57 | 321622 | hmgb1a |  |  | 31299 | -2.430 | -0.1236 | Yes |
| 58 | 393774\_2 | pole3 |  |  | 31836 | -4.872 | 0.0017 | Yes |
Table: GSEA details [plain text format]

  

Fig 2: BASE\_EXCISION\_REPAIR(DRE03410)      
 Blue-Pink O' Gram in the Space of the Analyzed GeneSet

  

Fig 3: BASE\_EXCISION\_REPAIR(DRE03410): Random ES distribution      
 Gene set null distribution of ES for **BASE\_EXCISION\_REPAIR(DRE03410)**
